# In utero choline exposure alters growth, metabolism, feed efficiency, and carcass characteristics of Holstein × Angus cattle from weaning to slaughter

**DOI:** 10.1093/jas/skad186

**Published:** 2023-06-12

**Authors:** William E Brown, Henry T Holdorf, Sara J Johnson, Sophia J Kendall, Sophia E Green, Heather M White

**Affiliations:** Department of Animal and Dairy Science, University of Wisconsin-Madison, Madison, WI, 53706, USA; Department of Animal and Dairy Science, University of Wisconsin-Madison, Madison, WI, 53706, USA; Department of Animal and Dairy Science, University of Wisconsin-Madison, Madison, WI, 53706, USA; Department of Animal and Dairy Science, University of Wisconsin-Madison, Madison, WI, 53706, USA; Department of Animal and Dairy Science, University of Wisconsin-Madison, Madison, WI, 53706, USA; Department of Animal and Dairy Science, University of Wisconsin-Madison, Madison, WI, 53706, USA

**Keywords:** beef, carcass characteristics, developmental programming, feed conversion

## Abstract

Feeding rumen-protected choline (**RPC**) to late gestation dairy cows has potential to affect growth in offspring. The objective of this study was to evaluate the effects of in utero choline exposure on the growth, feed efficiency (**FE**), metabolism, and carcass quality of Angus × Holstein cattle. Multiparous Holstein cows pregnant with male (*N* = 17) or female (*N* = 30) Angus-sired calves were enrolled 21 d prepartum and randomly assigned to one of four dietary treatments varying in quantity and formulation of RPC. The treatments included a control with 0 g/d supplemental RPC (**CTL**), supplemental RPC fed at the recommended dose (**RD**) of 15 g/d from either an established RPC product (**RPC1**_**RD**_; ReaShure; Balchem Corp.) or choline ion from a concentrated RPC prototype (**RPC2**_**RD**_; Balchem Corp.), or a high dose (**HD**) of RPC2 fed at 22 g/d (**RPC2**_**HD**_). From 2 to 6 mo of age, calves were group housed and offered 2.3 kg grain/hd/d (42% CP) with ad libitum grass hay, and stepped up to a complete finishing diet by 7 mo (12.0% CP; 1.34 Mcal/kg NE_g_). Weight and height were measured monthly. Animal FE was measured in individual pens for 35 d at 8 mo. Feed intake was measured daily, and blood was obtained on day 18 during the FE period. Afterwards, cattle were group housed and offered a free-choice finishing diet until slaughter, where carcass yield and quality characteristics were measured. Mixed models were used in PROC MIXED (SAS, 9.4) with the fixed effects of treatment, sex, time, their interactions, and the random effect of calf. Month was the repeated measure, and preplanned contrasts were used. Blood and FE data were analyzed with the fixed effect of dam choline treatment, calf sex, and the interaction. Increasing dose of RPC tended to increase weight over the entire study period. Feeding any RPC increased hip and wither height compared with CTL, and increasing RPC dose linearly increased hip and wither height. Treatment and sex interacted on DMI whereby increasing RPC intake linearly increased DMI for males but not females. Compared with control, feeding any RPC decreased plasma insulin, glucose, and an insulin sensitivity index (RQUICKI). In utero choline exposure increased kidney–pelvic–heart fat and marbling score. Mechanisms of action for intrauterine choline exposure on offspring growth, metabolism, and carcass characteristics should be explored as they have direct implications for profitability for cattle growers and feeders.

## Teaser

Beef × dairy calves exposed to choline in utero demonstrated increased growth and alterations in carcass quality indicators. In particular, an increase in marbling score with in utero choline exposure suggests prenatal choline exposure benefits beef × dairy cattle profitability potential.

## Introduction

Recent work in dairy cattle has demonstrated that a broad array of farm nutrition and management practices implemented for gestating dairy cows also positively affects offspring growth, health, and well-being (Laporta et al., 2017; Abuelo, 2020). Of particular interest is the use of rumen-protected choline (**RPC**), which is typically fed for 3 wk prepartum in the diets of dairy cows. Recent research showed that dietary RPC supplementation to prepartum dairy cows increased growth and feed efficiency (**FE**) of their preweaned and yearling offspring (Zenobi et al., 2018; Holdorf et al., 2023a).

Choline is a quasi-vitamin that is established as an essential nutrient in human nutrition (Zeisel and da Costa, 2009), but a requirement has not yet been determined in cattle. Choline functions as part of the cell membrane’s lipid bilayer as phosphatidylcholine, acts as the substrate for synthesis of the neurotransmitter acetylcholine, which is involved in neural signaling, and is a substrate for production of the methyl donor trimethylglycine. These three functions collectively influence offspring performance through placental, organ, and brain development, and through DNA methylation (Jiang et al., 2014; Zeisel, 2017). In utero choline exposure during late gestation is a potential nutritional intervention to enhance tissue growth and performance in the growing fetus since it is developing muscle and fat depots that are the basis for carcass growth and development later in life (Du et al., 2017). However, the timing, duration, and dose of in utero choline exposure that will optimally benefit the growing fetus and offspring in dairy cattle is not clear.

Enhancing calf growth through gestational mediators has value for calves destined for market purposes (i.e., beef production) since they are sold by weight and for carcass composition, depending on the age. The fact that advantages in growth and FE could be observed as late as 50 wk of age in dairy calves exposed to choline in utero (Zenobi et al., 2018) reveals long-term implications for growing cattle. There has been a recent rise in beef-on-dairy breeding strategies to capture more value for nonbreeding animals produced on dairy farms (Berry, 2021). Capturing more revenue through beef × dairy market calves can be an additional benefit from feeding RPC to peripartum dairy cows, since an effect of in utero choline exposure is possible. The objective of this study was to assess the long-term carryover effects of in utero choline exposure on the growth, metabolism, FE, and carcass characteristics of Angus × Holstein calves. We hypothesized that calves exposed to choline in utero would have greater gain and FE. The secondary objective of this study was to establish initial performance data on Angus × Holstein calves considering the paucity of published data for animals of this phenotype.

## Materials and Methods

The animal sampling and handling procedures utilized in this study were approved by the University of Wisconsin-Madison College of Agricultural and Life Sciences Institutional Animal Care and Use Committee (Protocol #A006338). Multiparous Holstein cows (*N* = 116) at the Emmons Blaine Dairy Cattle Research Center (Arlington, WI) were enrolled in the study 21 d prepartum as previously described (Holdorf et al., 2023a). Briefly, cows were randomly assigned to one of four dietary treatments varying in quantity and formulation of RPC. The treatments included a control with 0 g/d supplemental RPC (**CTL**), supplemental RPC fed at the recommended dose (**RD**) of 15 g/d from either an established RPC product (**RPC1**_**RD**_; ReaShure; Balchem Corp.) or choline ion from a concentrated RPC prototype (**RPC2**_**RD**_; Balchem Corp.), or a high dose (**HD**) of RPC2 fed at 22 g/d (**RPC2**_**HD**_).

Dairy cows were pregnant with either Holstein or Angus-sired calves. Only Angus-sired calves will be discussed in this paper (*N* = 47). Management of calves from birth to weaning has been described elsewhere (Holdorf et al., 2023a). Briefly, calves were fed 3.8 L thawed colostrum within 4 h of birth from a cow receiving the same dietary treatment as the calf’s dam. Thereafter, calves were relocated to the UW Beef Grazing Center (Arlington, WI) and were fed up to 0.8 kg DM milk replacer daily, split between two feedings, and offered ab libitum access to calf starter and water. Calves were housed individually until weaning at 53 d of age.

### Growth from weaning to 9 mo

Initial efforts sought to determine growth differences from 2 to 9 mo of age imparted by in utero choline exposure. After weaning, calves were group housed with access to ab libitum grass hay and offered 2.3 kg of a grain supplement daily until approximately 7 mo of age. As more calves were weaned, they were eventually split into two management cohorts of similar age. At approximately 7 mo of age, calves were relocated to the UW Beef Nutrition Center (Arlington, WI) and transitioned to a finishing diet. Nutrient composition of the feed ingredients and diets is outlined in Table 1.

**Table 1.** Nutrient composition of free-choice grass hay and protein supplement (approximately 2.1 kg/d) offered to Angus × Holstein calves from 2 to 8 mo of age. Calves were exposed in utero to choline through dam alimentation of different levels and formulations of rumen protected choline

	Ingredient	
Item	Grass hay	Protein supplement^1^
Chemical composition^2^		
DM, %	90.9	89.9
CP, %	14.5	16.3
NDFom, %	53.2	11.3
ADF, %	40.1	5.5
Starch, %	1.6	48.0
Ether extract, %	2.3	3.3
Ash, %	10.1	6.6
NE_G_, Mcal/kg	0.64	1.38

^1^Supplement ingredients on as-fed basis include ground corn (62%); soybean meal (14.5%); expeller soybean meal (5%); wheat mids (5%); soy hulls (4%); mineral premix (3.22%); molasses (5.1%); potassium chloride (0.2%); yeast (Diamond V; 0.63%); vitamin A (0.03%); vitamin D (0.01%); Vitamin E (0.15%).

^2^Reported as % of DM unless otherwise noted.

Individual calf weight, wither height, and hip height measurements were obtained monthly (30 ± 1 d) beginning at 5 mo of age. Calves were briefly restrained for all measurements in a chute equipped with electronic scales calibrated twice annually (Q-Catch 86-Series, Arrowquip, Woodlands, Manitoba; Silencer, Molly Manufacturing, Lorraine, KS).

### Feed efficiency and metabolism

To determine effects of in utero choline exposure on FE of Holstein × Angus calves fed a finishing diet, at approximately 8 mo of age calves were moved into individual pens for determination of FE over a 35 d period. The FE period was conducted in two cohorts. Calves were provided ab libitum access to water and a finishing diet containing 12% CP and 1.34 Mcal/kg NE_g_ (Table 2). Calves were fed once daily at 0800 hours, and total feed offered and orts were recorded. Samples of feed ingredients were obtained weekly. Feed samples were dried in a 55 °C forced air oven for 48 h and ground to pass through a 1 mm screen (Wiley Mill, Arthur H. Thomas). After grinding, weekly feed ingredient samples within cohort were composited on a weight basis for nutrient composition analysis using wet chemistry (Dairyland Laboratories, Arcadia, WI). Feed ingredients were analyzed according to the methods of Shreve et al. (2006) for DM. The content of crude protein (method 990.03), neutral detergent fiber (method 2002.04), acid-detergent fiber (method 973.18), lignin (method 973.18), ether extract (method 920.39), and ash (method 942.05) were determined according to AOAC International (AOAC International, 2016). Starch in feed and feces was analyzed utilizing a method undergoing AOAC collaborative review (30th Annual MW AOAC Meeting) and quantified on a YSI 2700 Select Biochemistry Analyzer (YSI, Inc.; Yellow Springs, OH). A single fecal grab sample was obtained from each animal for starch analysis to broadly assess the impact of whole-kernel high moisture corn passage in the feces. Furthermore, the high moisture corn mean geometric particle size was determined (ASABE, 2017) using a particle separator with 4,699, 2,380, 1,180, 589, 300, 150, and 63 µm sieves (W.S. Tyler Company, Mentor, OH).

**Table 2.** Dietary ingredients and nutrient composition of finishing diets offered to Angus × Holstein calves at approximately 8 to 9 mo of age. Calves were exposed in utero to choline through dam alimentation of different levels and formulations of rumen protected choline

	FE Cohort	
Item	1	2
Ingredients^1^		
Corn silage	26.0	25.4
High moisture corn	53.0	53.3
DDGS^2^	17.1	17.2
Mineral supplement^3^	0.04	0.04
Chemical composition^1^		
DM, %	72.6	71.2
CP, %	11.8	12.2
NDFom, %	18.4	19.7
ADF, %	13.2	9.8
Starch, %	51.0	49.7
Ether extract, %	4.7	4.6
Ash, %	4.7	4.6
NE_G_, Mcal/kg	1.34	1.34

^1^Reported as % of DM unless otherwise noted.

^2^Dried distillers’ grains with solubles.

^3^Supplement ingredients on an as-fed basis include: distillers’ grains (53%), calcium carbonate (23.5%), urea (6.4%), iodized salt (5.9%), Rumensin (5.0%), trace mineral premix (2.2%), potassium chloride (2.0%), selenium premix (1.1%), vitamin A (0.38%), thiamin (0.25%), vitamin E (0.25%), vitamin D (0.13%).

Calf weights were determined three times at the beginning, middle, and end of the feed intake data collection period, for a total of 9 body weight (**BW**) measurements over the course of the 35 d FE period. Average daily gain (**ADG**) was determined by fitting a regression of BW against the day of study. Total BW gain was calculated as the difference between the beginning and end BW based on the ADG regression. Feed conversion ratio (**FCR**) was calculated as the ratio of total DMI:gain during the 35 d study period.

Blood samples were obtained on day 18 of the FE period to assess in utero choline exposure on circulating metabolites while on a finishing diet. Blood was collected via coccygeal vessel venipuncture and evacuated into one tube containing potassium oxalate with a sodium fluoride glycolytic inhibitor and stored on ice until plasma separation (BD Vacutainer, Franklin Lakes, NJ). A serum tube with a clotting activator was used for serum collection after being allowed to sit at room temperature for 1 h (BD Vacutainer). Tubes were centrifuged at 2,000 × *g* (plasma) and 3,000 × *g* (serum) for 15 min, and the resulting plasma and serum were aliquoted and stored at −20 °C until analysis. Plasma insulin was quantified in triplicate using an enzyme-linked immunosorbent assay specific for bovine insulin (Mercodia, Inc., Uppsala, Sweden) and absorbance determined with a Synergy H1 spectrophotometer (Biotek Instruments, Inc., Winooski, VT). A CatachemWell-T autoanalyzer (Catachem, Inc., Oxford, CT) was used to quantify plasma glucose (C124-07), blood urea nitrogen (**BUN**; C264-04), and β-hydroxybutyrate (**BHB**; C442-02) in duplicate as described by Pralle et al. (2021). Serum triglyceride (**TG**) was also quantified in duplicate on the CatachemWell-T autoanalyzer, based on a 1:2 serial dilution standard curve of TG (Multi-calibrator lipids, FUJIFILM Wako Diagnostics, Mountain View, CA; Martin et al., 2021). Serum free fatty acids (**FA**) were quantified enzymatically with a modified plate assay on the Synergy H1 specrophotometer (Biotek Instruments) using Catachem reagents (C514-02; Pralle et al., 2021) and a 1:2 serial dilution standard curve of NEFA Standard Solution (FUJIFILM Wako Diagnostics). An internal control was included on each plate or run, with overall coefficient of variation less than 7.5% on all assays. The insulin sensitivity index (**RQUICKI**) was calculated according to the methods of Holtenius and Holtenius (2007) with the following equation:


$$\begin{array}{ll} RQUICKI= & \;1/[log\left(glucose\right)+log\left(insulin\right)\\ & +log\;\left(\;fatty\;acids\right)]. \end{array}$$

### Residual feed intake and relationship with growth and metabolic markers

Based on growth and intake data during the FE period, residual feed intake (**RFI**) was determined as the difference between the actual and predicted DMI. Predicted DMI was determined using multiple regression with the fixed effects of ADG, midpoint metabolic BW (**MBW**), and sex, and the random effects of dam choline treatment nested within FE cohort (JMP Pro 15, SAS Institute). Each animal’s RFI were then categorized based on deviation from the mean. Any RFI < 0.5 SD from the mean were considered low RFI (efficient; *N* = 15), and RFI > 0.5 SD from the mean were categorized as high RFI (inefficient; *N* = 17).

### Carcass composition

After the FE period, cattle were managed as a single group and offered a free-choice finishing diet with a self-feeder (Supplemental Table 1). Cattle did not receive an implant. The cattle were delivered to a beef processing facility (JBS Foods; Green Bay, WI) at approximately 16 mo of age in two separate cohorts aligning to the corresponding FE group. On the day before and day of delivery, cattle were weighed at 0700 hours before being transported to the facility. These BW were averaged before shipping and were used as the live weight for calculation of dressing percentage because individual weights were not obtainable at the slaughter facility.

Carcass traits were determined by the commercial processing facility. Hot carcass weight and kidney, pelvic, and heart (**KPH**) weight were determined on the day of slaughter. Dressing percentage was calculated as the hot carcass weight with KPH mass divided by the averaged live weight obtained prior to shipping. Approximately 24 h after slaughter, marbling score, ribeye area (**REA**), and USDA quality and yield grades were determined visually by a trained USDA grader working in the facility.

### Statistical analysis

Initial growth data were analyzed using repeated measures in SAS v 9.4 (PROC MIXED; SAS Inc., Cary, NC). Fixed effects included dam dietary choline treatment, calf sex, age, and their interactions. Management cohort was also included as a fixed effect but was removed in all analyses due to lack of significance (*P* > 0.10). Calf was included as a random effect, and month of age was specified as the repeated measure. Spatial power and autoregressive heterogeneous covariance structures were tested. A spatial power covariance structure was utilized in the final analysis based on a lower BIC and to account for unequal measurements between sampling timepoints. Calf birthweight was tested as a covariate in all models and removed when *P* > 0.10.

Statistical analysis from the FE period, as well as RFI and carcass data, were conducted using PROC GLIMMIX in SAS (9.4). Statistical models for each analysis are outlined below, and those independent variables lacking a coefficient represent random effects. The models were as follows:

FE period BW variables: Y = µ + ß_1_(dam choline treatment) + ß_2_(sex) + ß_3_(dam choline treatment × sex) + ß_4_(age covariate) + ß_5_(FE cohort covariate) + animal + residual error.

FE period remaining variables: Y = µ + ß_1_(dam choline treatment) + ß_2_(sex) + ß_3_(dam choline treatment × sex) + ß_4_(8 mo BW covariate) + ß_5_(FE cohort covariate) + animal + residual error.

RFI: Y = µ + ß_1_(RFI category) + residual error.

Carcass quality variables: Y = µ + ß_1_(dam choline treatment) + ß_2_(sex) + ß_3_(dam choline treatment × sex) + ß_4_(age covariate) + ß_5_(slaughter cohort covariate) + animal + residual error.

In all models, the fixed effect of FE cohort and covariates were removed from the models when *P* > 0.10. Preplanned contrasts were used to compare 1) CTL vs. RPC, 2) linear dose of RPC, and 3) quadratic response to RPC dose. Contrast co-efficients for the linear and quadratic contrasts were adjusted for uneven spacing of treatments using proc iml in SAS (v. 9.4). Externally studentized residuals were monitored for homogeneity of residuals to ensure all models met basic model assumptions, and dependent variables were transformed when appropriate. On some occasions, heterogeneous variances were modeled in order to meet model assumptions. A Tukey–Kramer adjustment was utilized to separate treatment means to avoid inflation of Type I error rate due to multiple comparisons. Data are presented with the least square means and 95% confidence intervals.

## Results

### Growth phase

**Figure 1.**
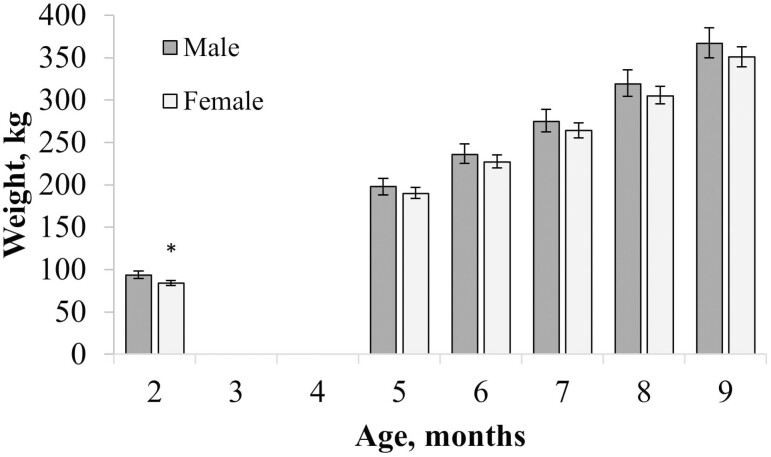
Interaction of time and sex for Angus × Holstein calf body weight (BW) (*P* = 0.09). Data are presented with 95% confidence intervals, and asterisk indicates significant difference (*P* < 0.05) in weight between sexes at a given timepoint.

There was a tendency for an interaction between sex and time (*P* = 0.09; Table 3), whereby males gained weight faster than females from 10 kg at 2 mo to 17 kg at 9 mo of age (Figure 1). There also tended to be a linear dose effect of RPC on calf weights (*P* = 0.10; Table 3) with greater RPC dose increasing calf weight.

**Table 3.** Effect of in utero choline exposure treatment, calf sex, and time on Angus × Holstein calf weight and dimensions from 2 to 9 mo of age presented as least square means and 95% CI

	Treatment^1^				Sex		*P-*value^2^				Contrasts^3^	
Item	CTL	RPC1_RD_	RPC2_RD_	RPC2_HD_	Male	Female	Trt	Sex	T	S × T	A	B
Weight, kg	214.5	220.9	228.6	225.2	216.4	228.3	0.28	0.05	<0.001	0.09	0.81	0.10
	[204.6, 225.0]	[207.2, 235.5]	[217.5, 240.3]	[215.3, 235.6]	[218.7, 238.3]	[210.1, 222.9]						
ADG, kg^4^	1.21	1.24	1.27	1.25	1.27	1.23	0.86	0.20	–	–	0.38	0.27
	[1.16, 1.27]	[1.17, 1.32]	[1.21, 1.34]	[1.19, 1.30]	[1.21, 1.31]	[1.19, 1.26]						
Withers, cm	104.9	106.8	107.2	106.6	105.1	107.6	0.14	0.01	<0.001	0.18	0.001	0.05
	[102.5, 107.3]	[104.1, 109.6]	[104.7, 109.7]	[104.1, 109.2]	[105.0, 110.3]	[100.1, 110.5]						
Hips, cm^5^	111.4^a^	113.0^a,b^	113.6^b^	112.6^ab^	113.5	111.8	0.07	0.02	<0.001	0.70	<0.001	0.05
	[110.3, 112.5]	[111.5, 114.6]	[112.4, 114.8]	[111.6, 113.7]	[112.3, 114.6]	[111.1, 112.6]						

^1^CTL = 0 g/d supplemental RPC; RPC1_RD_ = 15 g/d supplemental RPC (ReaShure; Balchem Corp.); RPC2_RD_ = 15 g/d supplemental RPC in concentrated prototype (Balchem Corp.); RPC2_HD_ = 22 g/d supplemental RPC in concentrated prototype (Balchem Corp.).

^2^ *P*-values for Trt × Sex, Trt × Time, Trt × Sex × Time were all *P* > 0.22.

^3^Contrast A: CTL vs. RPC; Contrast B: linear dose of RPC.

^4^ADG is from weaning to 9 mo.

^5^Quadratic effect for RPC dose (*P* = 0.09).

^a,b^Means within a row with different superscripts differ (*P* < 0.05).

Compared with CTL, RPC increased both wither and hip height (*P* ≤ 0.001; Table 3). There was also a linear dose effect of RPC for both wither and hip height (*P* = 0.05; Table 3), with increasing RPC dose increasing calf height. There tended to be a quadratic effect (*P* = 0.09; Table 3) of RPC dose for hip height, with hip height increasing from CTL to RPC_RD_ before decreasing slightly for RPC_HD_. Furthermore, there was a tendency for an overall treatment effect on hip height (*P* = 0.07; Table 3); RPC2_RD_ increased hip height compared with CTL (*P* < 0.05), but there was no evidence of difference between any other treatments.

### Feed efficiency period

There was an interaction between treatment × sex for DMI during the FE period (*P* = 0.02; Table 4), whereby RPC2 DMI was greater than CTL in male calves. There was no evidence of difference of treatment, sex, or their interaction on ADG (*P* ≥ 0.34; Table 4). There tended (*P* = 0.09; Table 4) to be an interaction between treatment × sex on FCR.

**Table 4.** Effect of in utero choline exposure on Angus × Holstein calf feed intake, gain, and FCR FE at approximately 8 to 9 mo of age presented as least square means and 95% CI

	Treatment^1^				Sex		*P*-value			Contrasts^2^	
Item	CTL	RPC1_RD_	RPC2_RD_	RPC2_HD_	Male	Female	Trt	Sex	Trt × Sex	A	B
Start BW, kg	356	362	364	366	372	353	0.77	0.03	0.44	0.87	0.29
	[341, 371]	[342, 384]	[348, 380]	[352, 381]	[358, 386]	[343, 362]					
End BW, kg	410	420	421	424	430	407	0.68	0.02	0.33	0.75	0.23
	[393, 428]	[396, 445]	[403, 440]	[408, 441]	[415, 446]	[397, 419]					
DMI, kg					–	–	0.16	0.60	0.02	0.68	0.37
Male	9.3^a^	10.2^a,b^	9.5^a,b^	10.4^b^							
	[8.9, 9.8]	[9.3, 11.2]	[8.9, 10.1]	[9.8, 11.0]							
Female	10.1	9.9	9.4	9.5							
	[9.6, 10.6]	[9.5, 10.4]	[9.0, 9.9]	[9.1, 10.0]							
ADG, kg	1.58	1.62	1.61	1.64	1.64	1.59	0.92	0.50	0.34	0.73	0.51
	[1.46, 1.71]	[1.45, 1.79]	[1.48, 1.74]	[1.52, 1.76]	[1.52, 1.75]	[1.51, 1.67]					
FCR					–	–	0.34	0.79	0.09	0.34	0.48
Male	6.13	6.93	5.76	6.25							
	[5.61, 6.66]	[6.07, 7.78]	[5.17, 6.36]	[5.75, 6.75]							
Female	6.56	6.01	6.20	6.11							
	[6.11, 7.00]	[5.59, 6.43]	[5.75, 6.65]	[5.63, 6.66]							

^1^CTL = 0 g/d supplemental RPC; RPC1_RD_ = 15 g/d supplemental RPC (ReaShure; Balchem Corp.); RPC2_RD_ = 15 g/d supplemental RPC in concentrated prototype (Balchem Corp.); RPC2_HD_ = 22 g/d supplemental RPC in concentrated prototype (Balchem Corp.).

^2^Contrast A: CTL vs. RPC; Contrast B: linear dose of RPC.

^a,b,c^Means within a row with different letters differ (*P* < 0.05).

Effects of in utero choline exposure and sex on blood metabolites at 8 mo of age are presented in Table 5. Dam choline intake affected offspring blood metabolites during the early finishing period. Exposure to any form of supplemental choline in utero decreased concentrations of plasma glucose and insulin, and increased the glucose:insulin (*P* < 0.01; Table 5). There was a similar linear response for increasing RPC dose on concentrations of glucose, insulin, and glucose:insulin ratio (*P* ≤ 0.02; Table 5). There was no evidence of a treatment difference for serum FA concentration (*P* = 0.31; Table 5). The RPC2_HD_ increased RQUICKI compared with all other treatments (*P* = 0.001; Table 5). Preplanned contrasts revealed that exposure to supplemental choline in utero increased RQUICKI compared with CTL (*P* < 0.001; Table 5), and RQUICKI increased with increasing dose of RPC (*P* < 0.001). Serum TG concentration tended to be less for calves exposed to RPC in utero compared with CTL (*P* = 0.09; Table 5). For plasma BHB concentrations, RPC2_HD_ increased BHB compared with CTL and RPC1_RD_ (*P* = 0.01; Table 5). There was a linear increase in plasma BHB concentration with increasing RPC dose (*P* = 0.02; Table 5). Males had a reduced concentration of BUN compared with females (*P* = 0.04; Table 5).

**Table 5.** Effect of in utero choline exposure on Angus × Holstein calf feed blood metabolites while on a finishing diet at approximately 8 to 9 mo of age presented as least square means and 95% CI

	Treatment^1^				Sex		*P*-value			Contrasts^2^	
Item	CTL	RPC1_RD_	RPC2_RD_	RPC2_HD_	Male	Female	Trt	Sex	Trt × Sex	A	B
Glucose, mg/dL	95.6	85.0	92.4	89.9	91.5	89.9	0.48	0.82	0.24	<0.01	0.02
	[91.3, 99.9]	[79.4, 90.7]	[88.0, 96.9]	[86.0, 94.0]	[87.7, 95.4]	[87.3, 92.5]					
Insulin, µg/L	4.4^a^	1.4^b^	3.1^a,b^	1.9^b^	2.3	3.0	0.01	0.24	0.28	<0.01	0.001
	[3.1, 6.0]	[0.5, 2.7]	[1.9, 4.5]	[1.3, 2.8]	[1.4, 3.3]	[2.3, 3.7]					
BUN, mg/dL	5.4	5.6	5.5	5.4	5.2	5.7	0.93	0.04	0.19	0.91	0.94
	[5.0, 5.9]	[5.0, 6.2]	[5.1, 6.0]	[5.0, 5.8]	[4.9, 5.6]	[5.5, 6.0]					
Fatty acids, m*M*	0.10	0.12	0.09	0.09	0.10	0.10	0.31	0.87	0.21	0.63	0.46
	[0.08, 0.12]	[0.09, 0.15]	[0.07, 0.11]	[0.07, 0.11]	[0.08, 0.12]	[0.09, 0.11]					
Triglyceride, mg/dL	18.1	14.6	16.0	16.0	16.2	16.1	0.49	0.95	0.27	0.09	0.20
	[15.2, 21.6]	[11.4, 18.6]	[13.3, 19.2]	[13.5, 18.9]	[13.8, 18.9]	[14.3, 18.0]					
BHB, m*M*	0.28^a^	0.24^a^	0.31^a,b^	0.37^b^	0.31	0.29	0.01	0.61	0.16	0.29	0.02
	[0.23, 0.32]	[0.18, 0.31]	[0.27, 0.36]	[0.33, 0.41]	[0.27, 0.35]	[0.27, 0.32]					
RQUICKI	0.34^a^	0.41^a^	0.38^a^	0.42^b^	0.40	0.38	0.001	0.28	0.46	<0.001	<0.001
	[0.36, 0.33]	[0.47, 0.36]	[0.42, 0.35]	[0.47, 0.39]	[0.43, 0.37]	[0.40, 0.36]					
Glucose:Insulin	21.6^a^	59.9^b^	29.6^a,b^	45.5^b^	40.5	30.5	0.01	0.22	0.29	<0.01	<0.01
	[16.0, 30.8]	[32.4, 146.0]	[20.7, 46.0]	[30.5, 75.1]	[28.1, 63.4]	[24.3, 39.4]					

^1^CTL = 0 g/d supplemental RPC; RPC1_RD_ = 15 g/d supplemental RPC (ReaShure; Balchem Corp.); RPC2_RD_ = 15 g/d supplemental RPC in concentrated prototype (Balchem Corp.); RPC2_HD_ = 22 g/d supplemental RPC in concentrated prototype (Balchem Corp.).

^2^Contrast A: CTL vs. RPC; Contrast B: linear dose of RPC.

^a,b,c^Means within a row with different letters differ (*P* < 0.05).

### Residual feed intake

Results for differences between efficient and inefficient beef × dairy calves fed with a finishing diet are in Table 6. Low RFI calves tended to have greater insulin (*P* = 0.08), but there was no evidence of difference for plasma glucose (*P* = 0.50) or serum FA (*P* = 0.44). The change in plasma insulin between groups corresponded to a tendency for a reduced glucose:insulin (*P* = 0.07; Table 6) and a reduced RQUICKI (*P* = 0.04) in low RFI calves compared with high RFI calves. Furthermore, BUN concentration was less in low RFI (*P* = 0.05; Table 6) compared with high RFI calves. There was no evidence of difference between efficiency classification for plasma BHB (*P* = 0.15; Table 6) or serum TG (*P* = 0.14). Furthermore, there was no evidence of difference for height or girth dimensions or dimension change over the FE study period (*P* ≥ 0.13; Table 6).

**Table 6.** Association between RFI classification and blood and body dimension measurements for Angus × Holstein calves at approximately 9 mo of age and fed a finishing diet, presented as least square means and 95% CI

	RFI^1^				
Item	Low		High		*P*-value
Glucose, mg/dL	92.6	[88.2, 96.9]	90.9	[88.1, 93.6]	0.50
Insulin, µg/L	3.5	[2.6, 4.7]	2.3	[1.5, 3.3]	0.08
BUN, mg/dL	9.6	[8.5, 10.9]	11.1	[10.3, 12.0]	0.05
Fatty acids, mEq/L	0.09	[0.07, 0.11]	0.11	[0.08, 0.13]	0.44
Triglyceride, mg/dL	18.8	[16.2, 21.6]	16.2	[13.9, 18.6]	0.14
BHB, m*M*	0.33	[0.30, 0.38]	0.29	[0.26, 0.33]	0.15
RQUICKI	0.36	[0.34, 0.37]	0.39	[0.36, 0.43]	0.04
Glucose:Insulin	23.3	[18.5, 31.8]	34.6	[25.7, 51.8]	0.07
Withers, cm	122	[119, 124]	122	[119, 124]	0.71
Hips, cm	129	[127, 132]	129	[127, 131]	0.84
Girth, cm	181	[176, 185]	181	[178, 183]	0.99
Withers change, cm	3.4	[2.4, 4.4]	4.7	[3.3, 6.0]	0.13
Hips change, cm	5.3	[3.7, 7.0]	4.8	[3.7, 5.9]	0.58
Girth change, cm	12.3	[10.2, 14.6]	10.9	[9.0, 12.9]	0.33

^1^Data are presented with 95% confidence intervals.

### Carcass quality

**Figure 2.**
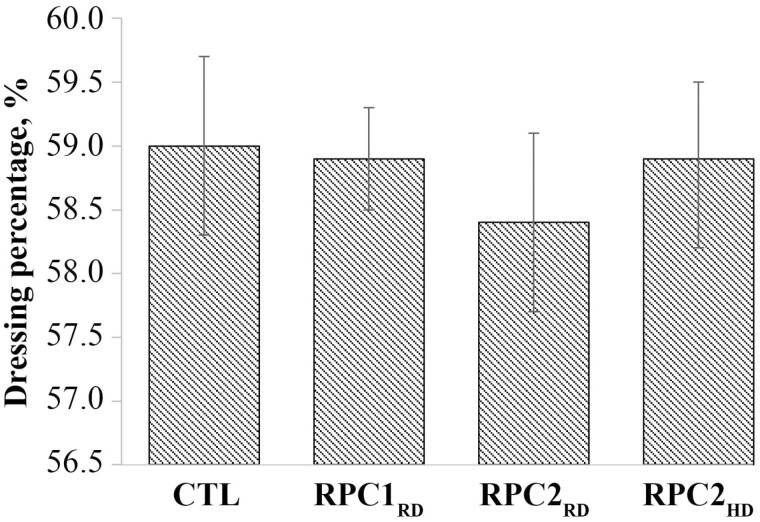
Effect of in utero rumen-protected choline exposure on Angus × Holstein dressing percentage (CTL vs. RPC *P* < 0.001). Data are presented with 95% confidence intervals. Treatments included: CTL = 0 g/d supplemental RPC; RPC1_RD_ = 15 g/d supplemental RPC (ReaShure; Balchem Corp.); RPC2_RD_ = 15 g/d supplemental RPC in concentrated prototype (Balchem Corp.); RPC2_HD_ = 22 g/d supplemental RPC in concentrated prototype (Balchem Corp.).

Males had a 60 kg slaughter weight and 36 kg hot carcass weight advantage compared with females (*P* < 0.001; Table 7), but there was no evidence of difference between RPC treatments for slaughter weight (*P* ≥ 0.83). Treatment and sex interacted for dressing percentage (*P* = 0.01; Table 7), whereby male dressing percentage was 2.2% less for RPC2_RD_ than RPC2_HD_, but there was no evidence of difference between treatments for females. Overall, exposure to RPC in utero decreased dressing percentage compared with control (*P* < 0.001; Figure 2).

**Table 7.** Effect of in utero choline exposure and sex on Angus × Holstein offspring carcass characteristics at approximately 16 mo of age, presented as least square means and 95% CI

	Treatment^1^				Sex		*P*-value			Contrasts^2^	
Item	CTL	RPC1_RD_	RPC2_RD_	RPC2_HD_	Male	Female	Trt	Sex	Trt × Sex	A	B
Slaughter	666	663	676	675	700	640	0.83	<0.001	0.54	0.94	0.31
weight, kg	[646, 687]	[626, 700]	[645, 709]	[656, 696]	[679, 721]	[627, 655]					
Hot carcass	393	393	394	397	412	376	0.98	<0.001	0.57	0.73	0.79
weight, kg	[378, 409]	[367, 418]	[372, 415]	[383, 411]	[398, 427]	[366, 385]					
Dressing percentage, %							0.55	0.59	0.01	<0.001	0.59
Male	58.9^ab^	59.0^a,b^	57.7^a^	59.9^b^							
	[57.9, 60.0]	[58.4, 59.6]	[56.5, 58.8]	[58.9, 60.9]							
Female	59.1	58.8	59.1	57.9							
	[58.2, 60.0]	[58.5, 59.1]	[58.2, 60.0]	[57.0, 58.7]							
KPH, kg	20.2^a^	25.0^b^	20.5^ab^	23.6^ab^	22.1	22.4	0.04	0.83	0.97	0.12	0.07
	[18.3, 22.2]	[21.9, 28.5]	[18.6, 22.7]	[19.5, 28.4]	[19.9, 24.4]	[20.7, 24.2]					
KPH, %	5.2^a^	6.4^b^	5.3^ab^	6.0^ab^	5.4	6.1	0.01	0.06	0.89	0.05	0.05
	[4.7, 5.7]	[5.8, 7.1]	[4.6, 6.0]	[5.0, 7.1]	[4.9, 5.9]	[5.6, 6.5]					
REA, cm^2^	93.5	85.8	91.0	90.3	89.0	91.0	0.66	0.70	0.90	0.11	0.26
	[87.1, 100.6]	[73.5, 99.4]	[83.2, 99.4]	[85.2, 95.8]	[82.6, 96.1]	[86.5, 94.8]					
Marbling	489	554	561	571	539	546	0.23	0.83	0.45	0.28	0.04
score^3^	[434, 550]	[473, 649]	[496, 634]	[511, 638]	[485, 599]	[508, 588]					

^1^CTL = 0 g/d supplemental RPC; RPC1_RD_ = 15 g/d supplemental RPC (ReaShure; Balchem Corp.); RPC2_RD_ = 15 g/d supplemental RPC in concentrated prototype (Balchem Corp.); RPC2_HD_ = 22 g/d supplemental RPC in concentrated prototype (Balchem Corp.).

^2^Contrast A: CTL vs. RPC; Contrast B: linear dose of RPC.

^3^Determined by USDA grader; small = 400 to 499, modest = 500 to 599, moderate = 600 to 699.

^a,b,c^Means within a row with separate letters differ (*P* < 0.05).

Treatment affected KPH weight (*P* = 0.04) and percentage (*P* = 0.01; Table 7). Compared with CTL, RPC1_RD_ increased KPH weight by 4.8 kg (1.2%; *P* < 0.05), but there was no difference between the other treatments. Furthermore, any RPC treatment increased KPH percentage compared with CTL (*P* = 0.05; Table 7), and increasing the dose of RPC linearly increased KPH percentage (*P* = 0.05) tended to linearly increased KPH mass (*P *= 0.07). Females tended to have greater KPH content than males (*P* = 0.06; Table 7). There was no evidence of treatment, sex, or interaction effects for REA (*P* ≥ 0.66; Table 7), but RPC treatments linearly increased marbling score (*P *= 0.04).

## Discussion

This data illustrate the long-term effects dairy management strategies can impart on growing calves. To date, investigation of most dairy maternal effects on offspring have been limited to the neonatal period up to weaning in heifer calves (Abuelo, 2020), but a growing body of evidence has illustrated a ­longer-term relationship between dairy dam management and offspring production performance. For example, dam body condition score, age at second calving, and milk somatic cell score are negatively associated with offspring milk and fat yields (Banos et al., 2007; Swartz et al., 2021), and exposing calves in utero to heat stress reduces first lactation milk production (Monteiro et al., 2016). While these effects should not be surprising considering that alterations in gestating beef cattle management have long-term implications on offspring growth, health and reproduction (Funston et al., 2010), it is inherently difficult in beef cattle research to separate gestational and lactational effects given that the calf remains with the dam. Therefore, dairy cattle represent a unique model since their offspring are separated from the dam at birth, enabling us to isolate effects of gestational environment on variables related to meat animal production.

This study also provides some of the first data on the performance of beef × dairy calves during the growing and finishing period in a North American production system (Foraker et al., 2022). Most of the existing literature evaluates performance of beef × dairy calves in grazing-based systems and utilizing beef sires not commonly used by North America dairies (i.e., Belgian Blue and Charolais; Berry, 2021). Therefore, this study is uniquely positioned to provide fundamental information on a growing and important segment of the North American beef and dairy industries.

### Growth through 10 mo of age

In this study, there was a tendency for an increase in BW from 2 to 10 mo of age with increasing dosage of dam dietary choline intake, but there was no evidence for a difference in ADG across treatments. Zenobi et al. (2018) reported an increase in BW at 50 wk of age in Holstein heifers exposed to choline in utero using 17 g of RPC, coupled with an increase in ADG from weaning to 50 wk. In contrast, supplementation of RPC to prepartum beef cows at a low dose (4.5 g/d; 60 d prepartum) failed to alter 205 d adjusted weaning weight of their calves compared with controls despite an increase in ADG at several stages before weaning (Pacheco et al., 2010). It is unclear why there are disparities in ADG and growth results across these three studies; however, multiple factors could play a role, including duration of maternal choline supplementation and stage of data collection, and environmental and management factors. Given that ADG or weight was positively affected in some manner across all three studies (and importantly was never negatively affected), additional investigation of offspring exposed to in utero choline will continue to provide a clearer picture as to when and for how long these advantageous effects may be realized under different management conditions.

The mechanisms by which in utero choline exposure may stimulate offspring growth are not clear, but choline’s role as a methyl donor may have contributed to altered DNA methylation in the calves in this study. Considering that any growth differences are likely manifested in greater muscle and adipose tissue depots in the offspring, a logical question is whether in utero choline exposure alters muscle or adipose DNA methylation. In whole blood collected on 3 d of age, global DNA methylation was increased in these male, but not female, calves exposed to choline in utero (Holdorf et al., 2023a); however, it is unclear if these patterns would be reflected in a tissue-specific manner. Work in the sheep fetus has shown that the dam’s diet influences muscle and adipose tissue DNA methylation gene expression (Lan et al., 2013; Namous et al., 2018), and increasing starch content in the diet of pregnant beef cows increased the gene expression of DNA methyltransferase 3 alpha in the *longissimus dorsi* muscle of neonatal calves (Wang et al., 2015). Supplementation of methyl donors may also influence the placenta, which could theoretically have long-term implications. For example, supplementation of choline in gestating mice increased global DNA methylation in the placenta (Kwan et al., 2018), and prepartum methionine supplementation in dairy cows upregulated placental DNA methylthransferase genes and altered markers of placental metabolism (Batistel et al., 2019). While the potential ­contribution of other methyl donors is important to consider, it is noteworthy that all dams in the present study were fed a diet that was supplemented with rumen-protected methionine to ensure that dietary methionine was sufficient and that any effect would be solely from choline supplementation.

The timing of choline supplementation and its effects on DNA methylation in tissues may also be pivotal, because the number of muscle fibers is determined by 7 mo of gestational age and any subsequent growth in muscle until birth and thereafter occurs only from hypertrophy (Du et al., 2010). Alternatively, adipose tissues are actively experiencing hyperplasia from midgestation to 250 d postnatally (Du et al., 2013) and may be more subject to dam nutritional changes during the final months of gestation in cattle than muscle tissue. Future work examining in utero choline exposure effects on DNA methylation in muscle and adipose tissues, coupled with exploration of choline supplementation for longer periods prepartum, is warranted.

There are other potential mechanisms that could have influenced the growth patterns in these calves. Choline supplementation decreased prepartum dam DMI and altered prepartum glucose and TG concentrations (Holdorf et al., 2023b). The lower DMI may have altered nutrient supply to the fetus in a manner that would have long-lasting effects after birth. Another potential explanation for the increased growth in calves exposed to choline while in utero may be from altered ruminal or hindgut microbiome. Changes in hindgut microbiome may aide in competitive exclusion of pathogens and help to maintain a healthy gut epithelium (O’Hara et al., 2020). This was seen previously when feeding prepartum Holstein cows methionine increased neonatal calf growth while altering the calf microbiome in a manner that may inhibit bacteria establishment (Elolimy et al., 2019). While these pieces of evidence may offer clues as to why growth patterns were altered in the calves on this study, more research in a variety of areas such as gene expression of tissues and characterizing rumen microbe communities is required to understand mechanisms by which choline could impart growth benefits on offspring.

In this study, there were clear advantages in frame height from 2 to 10 mo of age for calves exposed to RPC ­supplementation in utero compared with CTL. We had previously observed a quadratic relationship for height in the male calves in this study through 8 wk of age, whereby calf height was greatest at moderate levels of in utero choline exposure (Holdorf et al., 2023a). Sawant et al. (2019) administered RPC to ewes beginning at conception, which increased fetus femur and humerus length measurements compared to ewes not fed RPC via ultrasound on day 78 of gestation. While maternal undernutrition during gestation in ewes decreases lamb bone density and length (Reed and Govoni, 2017), very little is known about other aspects of gestational nutrition on bone formation in ruminants. Although we did not directly measure bone length in this study, given the height advantages in calves exposed to choline in utero, further work is needed to assess mechanisms by which in utero choline exposure could alter bone development and length in offspring.

### Dry matter intake and gross feed efficiency

There is evidence in rodents and humans that choline may affect molecules involved in satiety, specifically in the hypothalamus, and that this effect may carry over in offspring. The hypothalamus is a key control center of feed intake regulation, and the overall dam plane of nutrition has profound influence on offspring hypothalamic development and long-term feed intake control mechanisms (Bouret, 2012). In the current study, there was a sex-specific effect of maternal choline intake on feed intake, whereby male calves increased DMI during the FE period with greater dam choline intake, but there was no evidence of this difference in female calves. In mice, maternal choline consumption throughout gestation increased hypothalamic expression of neuropeptide Y neurons and leptin receptors in the pups at birth (Hammoud et al., 2020). Maternal mouse choline intake above recommended levels increased offspring cumulative food intake through 17 wk postweaning, increasing final BW in both sexes; however, differences between sexes were not reported (Hammoud et al., 2020). These findings raise the question of whether in utero choline exposure could have altered hypothalamic development in calves, because gestational choline supplementation in humans and rodents has positive effects on multiple aspects of neonatal brain development (Derbyshire and Obeid, 2020). We are not aware of any data evaluating the stage of hypothalamic development in the perinatal bovine, so it is unclear if choline supplementation under our treatment timeline could be directly influencing hypothalamic development. If future studies in cattle also reveal choline effects on feed intake after the neonatal period, evaluation of hypothalamic brain development may be useful in determining mechanisms of action.

There was no evidence of dam choline intake or sex effects on offspring ADG during the 35 d FE period. Dam dietary choline intake did increase FE in these calves through 8 wk of age, as previously reported (Holdorf et al., 2023a). Otherwise, data evaluating FE of offspring from dams supplemented with choline during gestation in cattle is nonexistent to our knowledge. Achieving sufficient power to detect differences in ADG and FE in individually-fed beef cattle is notoriously difficult (Richardson et al., 2004). Nonetheless, the study design met minimum feeding length (35 d) for accurate determination of FE in beef cattle (BIF, 2021), and compared with a 91 d test period, there is minimal improvement in the correlation coefficient for DMI, ADG, and FCR for feeding periods beyond 35 d in beef steers (Wang et al., 2006). Overall, more research is necessary to identify effects of in utero choline exposure on ADG and FE beyond the neonatal period.

The FCR ratio for the calves in this study ranged from 5.8 to 6.9 in males and 6.0 to 6.6 in females. Beef steers on a North American finishing diet typically have an FCR between 5.7 to 7.2 (Wang et al., 2006; Koenig et al., 2020), depending on the diet characteristics and age at which the FCR was determined. The early age at which these calves were placed on the finishing diet and measured for FE likely contributed to the positive FE performance (Duff and McMurphy, 2007). While there may be a perception that dairy × beef calves would be less efficient than their beef counterparts, there is little evidence for a difference in FE between beef and dairy genotypes and their crosses (Tjardes et al., 2002; Huuskonen et al., 2014; Hessle et al., 2019). Furthermore, high moisture corn in this study was primarily in whole-kernel form and particle size was comparatively large to achieve optimum starch digestibility and FE (5.6 ± 0.08 mm mean geometric particle size in Cohort 1; 5.8 ± 0.15 mm mean geometric particle size in Cohort 2). Fecal starch content in the diet herein is similar to that of others who fed dry rolled corn to feedlot steers (15% to 32%; Supplemental Table 2; Barajas and Zinn, 1998; Depenbusch et al., 2008). Decreasing particle size of grain decreases fecal starch content and increases apparent total tract starch digestibility (Beauchemin et al., 2001; Bengochea et al., 2005; Schwandt et al., 2016), and rolling high moisture corn increases FE compared with whole-kernel high moisture corn (Stock et al., 1991). In our study, changing the physical form of the corn grain source and the age at which FE was measured may have further enhanced FE.

### Metabolism effects

There were intriguing results for differences in metabolic control across treatments during the FE period. Plasma glucose and insulin decreased with increasing in utero choline exposure. Increasing choline dose also increased RQUICKI and glucose:insulin, which is indicative of greater insulin sensitivity. In the preweaning period for these calves, there was no evidence of difference in glucose or insulin metabolism (Holdorf et al., 2023a). However, studies in mice support our current findings where offspring from dams fed with a high fat diet supplemented with choline had greater insulin sensitivity and lower fasting glucose concentrations after weaning (Korsmo et al., 2020). There is also evidence that the influence of methyl donors on offspring metabolic control can occur as early as the peri-conception period. Rats from dams that were fed with methyl-deficient diets around conception had greater plasma insulin concentration and insulin resistance compared with controls (Maloney et al., 2011). While limited data is available in ruminants, the dam’s plane of nutrition, dietary energy source, and micronutrient composition have been demonstrated to alter glucose metabolism of offspring (Ford et al., 2007; Vonnahme et al., 2010; Radunz et al., 2012). The possibility of in utero choline exposure modifying glucose and insulin sensitivity in finishing cattle could have important consequences for carcass composition and profitability. For example, intramuscular fat, subcutaneous fat, and muscle tissues in cattle fed with a diet rich in gluconeogenic precursors respond differently to insulin in vitro (Rhoades et al., 2006), which may alter nutrient partitioning in a manner relevant to final carcass composition. The modification of apparent glucose and insulin sensitivity as a result of in utero choline exposure has implications for carcass quality in feedlot cattle, and should be explored.

Choline linearly increased plasma BHB concentration with increasing RPC dose, and the highest dose of RPC imparted greater plasma BHB than CTL. In postpartum dairy cows, RPC decreases circulating BHB (Arshad et al., 2020). However, the biology of the growing animal is different and the treatment differences observed in plasma BHB may be a function of alterations in rumen metabolism, potentially through increased butyrate production. A majority of ruminal butyrate is converted to BHB by the rumen epithelium, and continuous infusion of butyrate in the rumen of lactating dairy cows increases plasma BHB (Herrick et al., 2018). Coincidentally, the continuous infusion of butyrate also reduced plasma glucose concentration in lactating dairy cows (Herrick et al., 2018), which is consistent with the reduced glucose concentrations found in this study. It is also possible that in utero choline exposure altered gut microbial profile or epithelial function in a manner that would affect butyrate production, uptake, and conversion to BHB. For example, gestational supplementation of folate and choline in rats (Mjaaseth et al., 2021) and methionine in dairy cows (Elolimy et al., 2019) altered the colonic microbiome of the offspring around or after weaning. In the case of Mjaaseth et al. (2021), this corresponded to changes in the profile of colonic acetate and butyrate concentrations. At birth and at weaning, piglets that had been exposed to methyl donors in utero had more desirable intestinal morphology, increased enzyme activity, increased expression of nutrient transporter mRNA, and altered jejunal DNA methylation (Liu et al., 2017). It is unclear why in utero choline exposure increased plasma BHB in our study, but it may be related to alterations in ruminal metabolism or changes in intestinal epithelium function, and more work is needed to further evaluate this effect.

The plasma BUN concentrations were less in males than in females, which is corroborated by other researchers (Bailey et al., 2008; Walker et al., 2010). This may indicate greater nitrogen utilization from the diet to support muscle development and growth by males. Although there was no evidence of difference for ADG between sexes in this study, which would imply similar lean tissue deposition, this study may have been under powered to detect differences in ADG as discussed earlier.

### Residual feed intake

Evaluation of the relationship between variables of interest and RFI provide insight into the factors related to FE in livestock. This in turn offers an opportunity to understand potential management or genetic selection factors that may help to identify and enhance efficiency. While other studies have reported relationships between RFI and blood and frame measurements in growing beef cattle, the nature of heterosis induced by crossing beef and dairy cattle warrants investigation into whether there are differences in the relationship between performance factors and FE in beef × dairy crosses.

In our study, low RFI (efficient) calves had greater insulin concentration, and lower RQUICKI and glucose:insulin. This suggests that more efficient calves may have decreased insulin sensitivity, given the lack of difference in plasma glucose between RFI groups. Nascimento et al. (2015) also reported similar results for insulin and glucose:insulin between groups in growing Nellore bulls and heifers (250 kg). In another study, the relationship between RFI group and plasma insulin and RQUICKI differed depending on the type of diet offered to Charolais bulls (400 to 700 kg; Jorge-Smeding et al., 2021). Bulls fed the grass silage-based diet exhibited a similar response as in our study, but the bulls fed a higher-energy corn silage-based diet had an inverse relationship between RFI group and plasma insulin and RQUICKI (Jorge-Smeding et al., 2021). Insulin is negatively associated with the DMI response when cattle are fed a highly fermentable diet that provides abundant gluconeogenic precursors (Bradford and Allen, 2007). In fact, insulin can directly and indirectly reduce feed intake through both central and peripheral mechanisms (Roche et al., 2008), so it is possible that the elevated insulin concentration in more efficient calves actively created a negative feedback system to reduce feed intake.

The plasma BUN concentration was less for more efficient calves (low RFI), which is supported by data evaluating the relationship between blood metabolites and RFI in growing beef steers, heifers, and bulls (Fitzsimons et al., 2014; Nascimento et al., 2015; Jorge-Smeding et al., 2021). However, in Limousin × Holstein heifers, there was no evidence of difference in BUN between efficiency groups (Kelly et al., 2010). The lesser BUN concentration is expected with the efficient animals because less protein is consumed that can be deaminated in the rumen. Additionally, more efficient finishing cattle may have lower protein turnover combined with greater lean muscle mass as a proportion of total BW, resulting in greater overall nitrogen use efficiency (Jorge-Smeding et al., 2021).

There was no evidence of difference for the other blood metabolites analyzed in this study, which closely aligns with other studies in growing cattle (glucose, BHB, FA, TG; Fitzsimons et al., 2014; Clemmons et al., 2017; Jorge-Smeding et al., 2021). As it relates to circulating metabolites, it appears that insulin and BUN are the best overall indicators of FE in growing Angus × Holstein calves on a finishing diet.

Animal dimensions, including height and girth measurements, were assessed to determine their relationship with RFI. There was no evidence of difference for dimensions or dimension change over the FE period between efficiency groups. This is a common result in RFI studies in growing beef cattle (Nkrumah et al., 2004; Hafla et al., 2013; Parsons et al., 2020), and in Limousin × Holstein heifers (Kelly et al., 2010). Since animal dimensions are likely highly correlated with animal BW, the dimensions are likely partially captured in the DMI regression used to calculate RFI.

### Carcass characteristics

Our data reveal that in utero choline exposure in beef × dairy cattle impacts important carcass traits by increasing fat deposition in both internal and intramuscular fat depots. In contrast, feeding a very low dose of 4 g choline/d to gestating beef cows for 50 d prepartum failed to alter carcass characteristics of their offspring (Jaeger et al., 2009). Considering the low choline inclusion rate in that study, it may have been fed at a rate insufficient to alter performance.

With the importance of marbling in the beef industry, our results of an increase in marbling score for animals exposed to choline in utero are particularly intriguing. Intramuscular fat deposition is primarily manipulated through genetics, age, and feeding high-energy diets, and there are a lack of alternative nutritional methods or additives to actively enhance marbling. While there is some evidence that dam dietary restrictions during gestation in beef cattle may affect ­offspring marbling (Funston et al., 2012), the ability to delineate gestational and postnatal effects in those studies is difficult. Furthermore, the development of progenitor cells and active hyperplasia of adipose cells during the prenatal period (Du et al., 2010) represent an opportunity to influence dairy offspring characteristics via dam supplementation. While it remains unclear the specific mechanisms by which choline may be influencing adipose tissue development and metabolism, studies in cattle highlight that methyl donor exposure as early as the embryonic period alter DNA methylation in muscle and hepatic tissues (Jacometo et al., 2017; Estrada-Cortés et al., 2021), so perhaps adipose tissue is being programmed through methylation patterns for increased deposition. Alternatively, considering the apparent shifts in glucose metabolism in the calves exposed to choline in utero noted in the present study, the adipose tissue may have been more capable of utilizing circulating glucose for adipose tissue. The use of choline for the benefit of offspring marbling presents a promising opportunity, and further research is needed.

Overall, the dressing percentage in this study was low compared with industry standards, and likely reflects the fact that the animals experienced shrink between weighing and the time they were slaughtered. In regard to the effect of choline on dressing percentage, the in utero RPC exposure decreased dressing percentage compared with control. Surprisingly, KPH increased with RPC treatment, which is the opposite of what would be expected given the relationship between dressing percentage and KPH weight. This suggests that the internal organ, hide, or head mass was greater for animals with in utero choline exposure. Liu et al. (2017) noted enhanced intestinal morphology due to in utero choline exposure in pigs. Perhaps the in utero choline exposure promoted greater intestinal development and overall mass in offspring, resulting in the lower dressing percentage. However, the increase in KPH fat may be detrimental to profitability by decreasing the overall yield of boneless, retail cuts of meat. Conversely, another viewpoint is that in utero choline exposure could reduce the time of animals in the feedlot by achieving a high-quality retail product quicker based on intramuscular fat deposition alone.

This dataset also benefits livestock producers by making available one of the few peer-reviewed published datasets on carcass characteristics of Angus × Holstein cattle in North America despite Angus being the most commonly used beef sire breed on dairies (McWhorter et al., 2020; Pereira et al., 2022). Berry et al. (2021) reviewed beef × dairy carcass characteristics primarily in comparison to other breeds, but most cattle in their review were of European origin where the production practices and breed composition are vastly different from those utilized in North America. The fact that 98% of the cattle in this study achieved at least USDA grade Choice (including 15% Prime; Table 8) is an indication that Angus × Holstein cattle have potential as high-value contributors to the beef industry. In preliminary results from other institutions in the U.S., at least 75% of Holsteins crossed with Angus or Simmental also achieved USDA Choice or Prime, which was an improvement over their pure Holstein counterparts (Basiel et al., 2021; Dahlke and Harding, 2021; Foraker et al., 2022). Overall, this study indicates that beef × dairy breeding strategies is a useful mechanism to add value to the U.S. beef herd, yet more research is needed to understand factors that contribute to beef quality in beef × dairy cattle.

**Table 8.** Distribution of USDA yield and quality grade by in utero choline exposure treatment in Angus × Holstein cattle. Male and female data are combined

	Treatment^1^				
Item	CTL	RPC1_RD_	RPC2_RD_	RPC2_HD_	Overall Total
USDA yield grade					
3	10 (83%)	5 (50%)	6 (50%)	9 (69%)	30 (63%)
4	2 (17%)	5 (50%)	6 (60%)	4 (31%)	17 (37%)
USDA quality grade					
Prime	1 (8%)	2 (20%)	1 (8%)	3 (23%)	7 (15%)
Choice	10 (83%)	8 (80%)	11 (92%)	10 (77%)	39 (83%)
Select	1 (8%)	–	–	–	1 (2%)

^1^CTL = 0 g/d supplemental RPC; RPC1_RD_ = 15 g/d supplemental RPC (ReaShure; Balchem Corp); RPC2_RD_ = 15 g/d supplemental RPC in concentrated prototype (Balchem Corp.); RPC2_HD_ = 22 g/d supplemental RPC in concentrated prototype (Balchem Corp.).

## Conclusions

Feeding prepartum dairy cows RPC increased Angus × Holstein offspring BW and height through 10 mo of age. This nutritional intervention also enhanced indicators of insulin sensitivity when calves were fed a finishing diet, which could have implications for carcass composition. More efficient calves according to RFI classification had indicators of impaired insulin sensitivity, which could potentially provide a mechanism for feed intake differences between high and low efficient feeder cattle. In utero choline exposure increased kidney, pelvic, and heart fat deposition, but notably, resulted in a greater marbling score. Overall, an increase in growth in calves exposed to choline in utero illustrates the potential to use dam dietary choline supplementation to enhance performance and profitability of offspring destined for market purposes, and further investigation into the mechanisms of action responsible for these positive benefits is warranted.

## Supplementary Material

skad186_suppl_Supplementary_File

## Abbreviations

ADG: average daily gain
BHB: *beta*-hydroxybutyrate
BUN: blood urea nitrogen
BW: body weight
FA: fatty acids
FCR: feed conversion ratio
FE: feed efficiency
KPH: kidney–pelvic–heart weight
MBW: metabolic body weight
REA: ribeye area
RFI: residual feed intake
RQUICKI: insulin sensitivity index
RPC: rumen-protected choline
TG: triglycerides
