# Supplementary material for: In utero choline exposure alters growth, metabolism, feed efficiency, and carcass characteristics of Holstein × Angus cattle from weaning to slaughter
Source: J Anim Sci. 2023 Jun 12;101:skad186. doi: 10.1093/jas/skad186 (PMC10294555; doi:10.1093/jas/skad186)
Supplement: skad186_suppl_Supplementary_File [file skad186_suppl_supplementary_file.docx]

**SUPPLEMENTAL TABLES**

**Supplemental Table 1.** Dietary ingredients and formulated nutrient composition of finishing diets offered via a self-feeder to Angus x Holstein cattle at approximately 10 to 16 months of age. Calves were exposed in utero to choline through dam alimentation of different rates and formulations of rumen protected choline.

| Item |  |
| --- | --- |
| Ingredients^1^ |  |
| Whole corn | 19.9 |
| Cracked corn | 21.9 |
| Wheat midds | 44.8 |
| Molasses | 1.5 |
| Fat | 1.7 |
| Vitamin and trace mineral supplement^2^ | 10.3 |
|  |  |
| Chemical composition^1^ |  |
| DM, % | 89.5 |
| CP, % | 13.2 |
| aNDF, % | 23.7 |
| Ether extract, % | 5.8 |
| NE_G_, Mcal/kg | 1.32 |

^1^Reported as % of DM unless otherwise noted

^2^The supplement consists of 56.7% dry distillers’ grains, 21.7% calcium carbonate, 14.9% cracked corn, 2.8% Rumensin, 1.8% iodized salt, 0.83% urea, 0.83% vitamin E (9.1 IU/g), 0.42% trace mineral premix, 0.08% vitamin A (6.2 IU/mg), and 0.03 vitamin D (1.8 IU/mg)

**Supplemental Table 2.** Fecal starch concentration of Angus x Holstein calves fed a finishing diet at approximately 9 mo of age. Data are displayed by sex and in utero choline exposure treatment.

| Item^1^ | Mean | SD^2^ |
| --- | --- | --- |
| Male |  |  |
| CTL | 14.4 | 5.30 |
| RPC1_RD_ | 25.0 | 8.63 |
| RPC2_RD_ | 17.4 | 10.96 |
| RPC2_HD_ | 32.0 | 9.26 |
| Female |  |  |
| CTL | 19.8 | 1.77 |
| RPC1_RD_ | 19.0 | 0.07 |
| RPC2_RD_ | 15.8 | 4.45 |
| RPC2_HD_ | 24.7 | 4.45 |

^1^CTL = 0 g/d supplemental RPC; RPC1_RD_ = 15 g/d supplemental RPC (ReaShure; Balchem Corp); RPC2_RD_; 15 g/d supplemental RPC in concentrated prototype (Balchem Corp); RPC2_HD_ = 22 g/d supplemental RPC in concentrated prototype (Balchem Corp)

^2^Standard deviation
